# Effect of Gender and Physical Activity on Internet Addiction in Medical Students

**DOI:** 10.12669/pjms.331.11222

**Published:** 2017

**Authors:** Muhammad Alamgir Khan, Faizania Shabbir, Tausif Ahmed Rajput

**Affiliations:** 1 Dr. Muhammad Alamgir Khan, FCPS. Professor of Physiology, Army Medical College, Rawalpindi, Pakistan; 2 Dr. Faizania Shabbir, FCPS. Assistant Professor of Physiology, Rawalpindi Medical College, Rawalpindi, Pakistan; 3 Dr. Tausif Ahmed Rajput, PhD. Professor and Chairperson, Shifa Tameer-e-Millat University, Islamabad, Pakistan

**Keywords:** Internet addiction, Young’s internet addiction test

## Abstract

**Objective:**

To determine the effect of gender and physical activity on internet addiction in medical students.

**Methods:**

In this cross sectional, analytical study Young’s internet addiction test questionnaire was distributed to 350 MBBS students of Army Medical College, Rawalpindi. The study was conducted from January to May 2015. A dichotomous response from students regarding physical activity was obtained which was verified from the sports department of the institution. Based upon total score, internet addiction was categorized as no addiction if the score was less than or equal to 49, moderate addiction when the score was 50 to 79 and severe when the score was 80 to 100.

**Results:**

Out of 322 respondents 175 (54.3%) were males and 147 (42.7%) females with a mean age of 19.27±1.01 years. Total internet addiction score and frequency of internet addiction were similar between males and females (37.71±11.9 vs 38.63±14.00, p=0.18 and 25 vs 29, p=0.20). However, total score and frequency of internet addiction were higher in students lacking physical activity as compared to those with regular physical activity (40.37±15.05 vs 36.38±11.76, p=0.01 and 30 vs 24, p=0.01).

**Conclusion:**

Internet addiction is unrelated to gender however it is inversely related to physical activity.

## INTRODUCTION

Internet is a source of knowledge, latest information, communication and entertainment. The background purpose of internet was to facilitate communication and promote research, however excessive self-indulgence and pathological use of this medium has led to development of a proper term that is internet addiction disorder. Internet addiction disorder (IAD) is defined as “one’s inability to control internet using, which could lead to physical, psychological, and social difficulties”.^1^ The suggested activities that are particularly addictive on internet are online gaming, online shopping and social networking.^2^

Internet usage has increased explosively, with greater than 3.5 billion users worldwide as recorded on first July 2016.^3^ Availability of internet facility in portable devices like smart phones and tablets is one of the major contributing factor towards its excessive use. It has become an indispensable part of current modern living.^4^ The young generation has seen this facility from birth, is accustomed to its 24-hour access and cannot think of accomplishment of their daily tasks in its absence.^5^ Youth and particularly college going young adults are at an increased risk of problematic internet use due to free internet access at college campuses and absence of strict parental supervision. Excessive internet usage in university students results not only in psychological but physical problems which leads to deterioration in their academic performance.^6^ In the modern life style where technology including internet has facilitated man, it can be a possible cause of reduced physical activity.^7^ It is a general observation that students who are actively involved in physical activity are less interested in sedentary activities like sitting on internet for long hours, however, empirical research on this topic is scant.

Although internet usage is common among both genders and all age groups, adolescents and young people are particularly vulnerable.^8^ The increased prevalence in adolescents is due to the fact that they are in the process of developing psychologically, emotionally unstable, less self regulative, easily susceptible to media influence and vulnerable for developing addictive behaviour.^4^ Internet has penetrated so deeply into our lives that we cannot exclude it from our day to day activities just on the pretext that it has some bad effects. There is a huge amount of academic stuff available on internet and the students just cannot think doing their academic activities without internet whereas on the other end of spectrum are the serious problems like internet addiction. This baffled scenario requires evidence based upon empirical research about internet addiction and different variables related to medical students and their academic performance. The present study was an attempt to determine the effect of gender and physical activity on internet addiction in medical students of Army Medical College, Rawalpindi.

## METHODS

This cross sectional analytical study was conducted from January to May 2015 at Army Medical College, Rawalpindi after getting approval from Ethical Review Committee of the institution vide letter number 02/CREAM/-A/Alamgir.

Male and female students of MBBS classes were selected by non-probability convenience sampling who gave written informed consent to participate in the study. Students who did not have computer or related device (like Smart phone, Laptop, Tablet) were excluded. Sample size was calculated using WHO sample size calculator. By keeping the values of confidence level, anticipated population proportion and absolute precision as 95%, 0.25 and 0.05, a sample size of 289 was calculated. Data collection tool used in the present study was ‘Young’s Internet Addiction Test’ questionnaire developed in 1998 by Young.^9^ This is a fully validated, self-administered questionnaire comprising of 20 closed ended items with responses on 5 points Likert Scale. At an expected response rate of about 85%, the questionnaires were distributed to 350 MBBS students. However, completely filled questionnaires were returned by 322 students. Based upon total score, internet addiction was categorized as no addiction if the score was less than or equal to 49, moderate addiction when the score was 50 to 79 and severe when the score was 80 to 100. Physical activity was defined operationally as any activity performed daily for at least one hour that resulted in energy expenditure by using skeletal muscle like walking, running and playing specific sports. A dichotomous response from students regarding physical activity was obtained which was verified from the sports department of the institution.

Data was analyzed using SPSS version 23. For numerical values like total internet addiction score and age, mean and standard deviation were calculated, and for categorical variables like gender, frequency and percentages were calculated. Total internet addiction score was compared between the two groups (male vs female and physical activity vs no physical activity) using independent samples t test. Frequency of students with internet addiction was compared across gender and physical activity groups using Chi Square test. Multiple linear regression was used to determine the combined effect of gender and physical activity on total score of internet addiction. Alpha value was kept at 0.05.

## RESULTS

There were 175 (54.3%) male and 147 (42.7%) female participants (N=322) with a mean age of 19.27±1.01 years. Reliability of the instrument as determined by Cronbach’s Alpha was found to be 0.90.

The total internet addiction score of females was 38.63±14.00 whereas for males it was 37.71±11.99. The difference of the score between males and females was statistically insignificant at p-value of 0.18. Likewise, the total internet addiction score for students who were involved in regular physical activity was 36.38±11.76 whereas for the students with no physical activity it was 40.37±15.05 and the difference was statistically significant at p-value of 0.01.

Two hundred and sixty-eight students (83.2%) had no internet addiction, 52 (16.1%) had moderate and 2 (0.6%) students had severe internet addiction. Out of 322 students, 54 (16.7%) had mild to moderate internet addiction.

Frequency of students with internet addiction was compared between males and females to find out effect of gender on internet addiction as shown in Table-I. The difference in frequency was statistically insignificant at p-value of 0.2. Similarly, frequency of students with internet addiction was compared between those with and without physical activity to find out effect of physical activity on internet addiction, also shown in Table-I. The difference in frequency was statistically significant at p-value of 0.01.

**Table-I:** Frequency comparison of internet addiction across gender and physical activity groups.

		Internet addiction		p-value

		Yes	No
Gender	Male	25 (46.3%)	150 (56%)	0.20
	Female	29 (53.7%)	118 (44%)	
Physical activity	Yes	24 (44.4%)	194 (72.4%)	0.01*
	No	30 (55.6%)	74 (27.6%)	

* P-value significant (<0.05)

Combined effect of gender and physical activity on internet addiction was calculated using multiple linear regression and is shown in Table-II. The results show that physical activity significantly affect internet addiction score even after controlling for gender (p-value=0.03).

**Table-II:** Multiple linear regression analysis of combined effect of gender and physical activity on internet addiction.

Independent variable	Standardized coefficients (Beta)	t-value	p-value
Gender	0.02	0.24	0.81
Physical activity	0.14	2.2	0.03*

* P-value significant (<0.05)

## DISCUSSION

In current study, effect of gender and physical activity on internet addiction was explored using two statistical perspectives. One is, comparing total internet addiction score between males/females and those with and without physical activity and the other is comparing frequency of students with internet addiction across the two genders and physically active/inactive groups. This ‘triangulation approach’ enhanced reliability of the results.

The mean internet addiction score was statistically similar across the two genders (p-value=0.18) although the mean score was slightly higher in females as compared to males. The same results were achieved when frequency of students with internet addiction was compared between males and females. Although frequency of female students with internet addiction was higher as compared to males, however, the difference in frequency was statistically insignificant (p-value=0.20). Regarding the effect of gender on internet addiction, various studies have generated inconsistent results. Shek et al. conducted a study in high school adolescents of Hong Kong and found that internet addictive behavior was consistently high for male gender.^10^ Chiu et al. conducted a study on internet addiction through smart phones in college students of Taiwan and found that female students were more addicted than male students.^11^ Malik et al. conducted a research on students of University of Sargodha, Pakistan to study Facebook addiction and found no gender predominance.^12^ Fernandez et al. investigated problematic internet use among university students and found that students of other than health sciences spent more time surfing internet however, there was no difference regarding gender.^13^ The results of study conducted by Alavi et al. at among students of Isfahan’s universities showed that males were at three times greater risk of developing internet addiction than females.^14^ The varied results regarding gender differences and internet addiction can be attributed to multiple factors like cultural values, access to internet, institutional policies and personal habits etc.

The mean internet addiction score was significantly higher in students who were not involved in any kind of physical activity as compared to those who were involved (p-value=0.01). Similar results were found when frequency of students with internet addiction was compared between those with and without physical activity. Frequency of internet addiction was significantly higher for students who lacked any physical activity as compared to those who were in the habit doing physical activity (p-value=0.01). Students who take part in any kind of physical activity tend to stay away from gadgets that use internet. They are more inclined towards healthy activities instead of spending time on internet. They tend to sleep early because of physical tiredness so chances of internet usage till late night are rare in these students. On the other hand, students who do not participate in physical activities are lazy and tend to remain stuck with internet gadgetries.^15^ Warbrick et al. conducted a study to find out the factors that result in reduced physical activity in natives of New Zealand and found technology as a major cause of distraction. Due to intense revolution in technology, indoor activities have replaced outdoor activities “Now they’ve got machines and buttons and that seems to be the recreational pathway at the moment”.^16^ Sarah Spengler et conducted a study on media use and physical activity and found results consistent to those of our study. They found that two third of adolescent population was engaged in both media use and physical activity but one activity was predominant. Results of this study supported the hypothesis that there is a competition between physical activity and media use.^17^ A multicenter study with larger sample size is recommended to be carried out to find out the effects of gender and physical activity on internet addiction so that the results can be extrapolated on to the general population with reasonable confidence level.

## CONCLUSION

Internet addiction is unrelated to gender however it is inversely related with physical activity. Students who appear to be involved in internet addiction may be advised to participate in any kind of physical activity which may lead to drop in their internet addiction level.
